# Oral Administration of Acrylamide Worsens the Inflammatory Responses in the Airways of Asthmatic Mice Through Agitation of Oxidative Stress in the Lungs

**DOI:** 10.3389/fimmu.2020.01940

**Published:** 2020-10-09

**Authors:** Bahador Hajimohammadi, Seyyede Masoume Athari, Mohammad Abdollahi, Ghasem Vahedi, Seyyed Shamsadin Athari

**Affiliations:** ^1^Research Center for Food Hygiene and Safety, Shahid Sadoughi University of Medical Sciences, Yazd, Iran; ^2^Department of Biology, Faculty of Basic Sciences, Maragheh University, Maragheh, Iran; ^3^Toxicology and Diseases Group, Pharmaceutical Sciences Research Center, Tehran University of Medical Sciences, Tehran, Iran; ^4^Department of Toxicology and Pharmacology, Faculty of Pharmacy, Tehran University of Medical Sciences, Tehran, Iran; ^5^Department of Immunology, School of Medicine, Zanjan University of Medical Sciences, Zanjan, Iran

**Keywords:** acrylamide, food contamination, allergic asthma, toxicology, oxidative stress

## Abstract

Acrylamide is a toxic chemical substance produced when starch-rich foods are fried at high temperatures. Asthma is a chronic and complicated respiratory disease, of which genetic and environmental factors are the main triggers. Orally-received components may have an effect on asthma pathophysiology. The aim of this study was to investigate the role of AA as a stimulus in asthma. BALB/c mice were allocated into four groups as follows: two OVA-sensitized asthmatic groups, including one treated with AA by gavage feeding and one non-treated (asthma group), and two healthy (non-asthmatic) groups, one treated with AA by gavage feeding and one non-treated (negative control group). Airway hyperresponsiveness, cell count, cytokine levels in BAL fluid, lung histopathology, IgE levels, and oxidative stress indices including plasma level of MDA, pulmonary antioxidant enzymes (SOD and CAT) levels, HP content, and collagen fiber accumulation in lung tissue were measured. We found that the group of mice treated with both OVA and AA (asthmatic and AA-treated mice) experienced higher levels of asthma-associated biomarkers, including higher enhanced pause (Penh value), eosinophilic inflammation, mucus hyper secretion, goblet cell hyperplasia, total and OVA-specific IgE levels, IL-4, IL-5, and IL-13 levels than the group sensitized only with OVA (asthmatic mice). The OVA-AA-treated mice also experienced worsened levels of oxidative stress indicators. Healthy (non-asthmatic) mice that only received AA were in similar conditions to healthy untreated mice (negative control group). The OVA-AA-treated group showed more severe allergic asthma symptoms in comparison to the group only sensitized with OVA. Therefore, food/water contaminated with AA can act as a stimulant of allergic asthma and exacerbate the bronchial inflammatory responses.

## Introduction

Acrylamide is a toxic chemical substance found in baked and fried foods such as potato chips, cookies, and biscuits. When starch-rich foods are fried at high temperatures, AA is formed in the food. Asparagine is one of the main precursors of acrylamide formation (1). AA is formed in the presence of the amino acid asparagine and compounds with carbonyl moiety, such as starch. AA arises at temperatures above 120°C (2, 3).

AA has toxicity effects on tissues and causes irritation of the skin, eyes, and airways. AA toxicity results from dermal absorption rather than from inhalation routes (4–6). Pervious evidences showed that AA derived during food processing may induce neurotoxic, genotoxic, and carcinogenic effects. AA has also been classified as a potential human carcinogen. In addition, dietary exposure of AA can increase health risks and some diseases' severity. Therefore, considering the increasing consumption of heat-processed foods, the potentially harmful effect of AA on human health should not be ignored (7–10). AA is also found in cigarette smoke, which is a major way smokers are affected, and cigarette smoke is an important trigger of asthma in predisposed patients (11, 12).

Asthma is one of the main complex syndromes of the respiratory system and is characterized by recurrent episodes of coughing, dyspnea, chronic airway narrowing, wheezing, increased bronchial responsiveness to inhaled stimuli, airway eosinophilic inflammation, mucus hyper secretion, and recurrent smooth muscle spasm. Asthma is a multifactorial disease controlled by genetic and environmental factors (13, 14). Allergic asthma is mediated by IgE, which is induced by allergens and produced from plasma cells under force of Th2 (Type-2) cytokines. The tendency to produce IgE is a genetically determined malfunction in asthmatic patients. Once produced, IgE binds to mast cells and therefore a subsequent re-exposure to an allergen can cause the formation of the antigen (allergen)-antibody complex on the surface of the mast cells, which triggers both the release of the stored mediators in the cytoplasmic granules and the synthesis and release of other mediators that themselves lead to further heightening of airways' inflammation and bronchoconstriction. Type-2 cytokines are produced by Th2 cells, which have a critical role in the pathophysiology of allergies and asthma. The prevalence of asthma has increased over recent decades because of undesired changes in lifestyle and environmental factors, particularly in urban areas (15–17). Currently, available drugs cannot completely cure asthma and the control of asthma attacks is a considerable challenge in the world. Allergen exposure is the main stimulant of asthma, so the prevention of exposure of patients to allergens and triggers is important in the management and control of asthma (18, 19). In the present study, we planned a simulation of AA-contaminated food by oral administration of AA in a murine model of asthma. The aim of the study was to investigate the possible harmful impact of AA on the exacerbation of immunological, serological, and histological symptoms of allergic asthma.

## Materials and Methods

### Animal

Six-week-female BALB/c mice (with a mean body weight of 19 g) were purchased from the Pasteur Institute (Karaj, Iran) and acclimatized to the standard conditions (24 ± 2°C, 50 ± 10% humidity, 12 h light-dark cycle and pathogen-allergen free) by being maintained in the animal room for 1 week before starting the experiments.

### Animal Sensitization, Treatment Schedule, and Sacrificing Method

**Figure 1:**
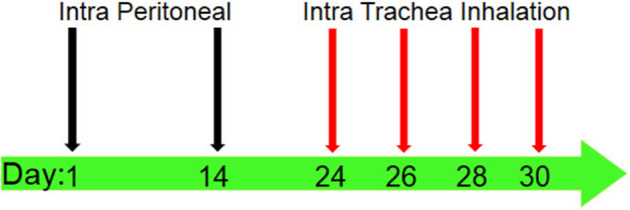
Sensitization and challenge protocol for animal asthma model producing. Mice were immunized by intraperitoneal (IP) injection of 20 μg OVA (which was solved in 30 μl normal saline) with 1 mg alum adjuvant at days 1 and 14. The sensitized mice were challenged by intra trachea inhalation (IT) with 1% OVA solution aerosolized by ultrasonic nebulizer for 30 min per day in days 24, 26, 28, and 30 to produce asthmatic model.

Ninety-six mice were allocated in to four groups (*n* = 24). In two groups, airway inflammation was induced by ovalbumin (Sigma-Aldrich, USA) (OVA-sensitized) according to a previously described protocol (20–22) (Figure 1). To induce the murine model of allergic asthma, the mice were sensitized by intraperitoneal injection of 20 μg chicken OVA and 1 mg aluminum hydroxide (Sigma-Aldrich, Netherlands) (Alum) as an adjuvant dissolved in 30 μl normal saline at day 1. Also, a subsequent boosting injection of OVA was applied at day 14. Next, at days 24, 26, 28, and 30, the mice in two OVA-injected groups inhaled 1% OVA aerosols for 30 min/day (aerosolized by ultrasonic nebulizer NE-U07, Omrom, Japan). Two remaining groups of mice were not sensitized. Also, one of the OVA-sensitized groups received AA by oral administration of AA-containing water. One other asthmatic group received a normal diet (without AA) (asthmatic control group) (23). In addition, one of the two healthy non-asthmatic groups was gavage-fed with the AA-containing water daily (in a similar way to asthmatic group that received AA). The other healthy group received a normal diet without AA (healthy negative control). AA treatment started at day 30 and continued for 8 weeks (until day 86). AA was administered at a constant dose of 2 mg/kg of feed/diet per day, which was equal to daily administering of 10 μg of water-dissolved AA per mouse. The administered dose was based on considering the mean daily consumption of 5 gr of diet. Therefore, the mice received AA 10 μg/per day which was dissolved in 0.1 ml of ultrapure water (Merck, Darmstadt, Germany), and the daily administration continued over the next 8 weeks. AA dose was chosen according to published toxicology studies (24), which claim the highest content of AA is in potato crisps (about 2,000 μg/kg = 2 mg/kg). We also considered the highest daily intake of LD_50_ and BMDL_10_ (24) for the selection of a proper test dose of AA. The samples were collected at weeks 2 (day 44), 4 (day 58), 6 (day 72), and 8 (86), each time by scarification of 1/4 of the mice. The samples (BAL, blood, lung tissue) were investigated for histopathology, IgE levels, cytokines levels, eosinophils (Eos) count in BAL, oxidative stress biomarkers, and the footprints of fibrotic changes (i.e., pulmonary hydroxyproline and TGF-β level). The blood samples were collected from the tail vein of all mice. For sacrificing the mice, an injection cocktail of ketamine (100 mg/kg) and xylazine (10 mg/kg) was used. Lungs of mice were used to prepare lung tissue homogenates.

### Collection of BAL Fluid

At the end of every 2 weeks after starting AA-treatment, BAL fluid was collected from the trachea after lavage of the airways with 1 ml of PBS. Collected BAL fluid was cytospined and stained with Wright-Giemsa, and then the cells were counted. The supernatant of centrifuged BAL fluid was stored at −70°C for further cytokine measurements.

### Airway Responsiveness to MCh (MCh Challenge Test)

The airway hyperresponsiveness (AHR) was measured by methacholine challenge a day before each instance of sacrificing the mice (at days: 43, 57, 71, and 85) in the manner described earlier (20–22). AHR was assessed by determining enhanced pause (Penh value). Mice were anesthetized and then tracheotomized after reaching a stable condition. A catheter was placed in the trachea and then connected to a mechanical ventilator (Inspira ASV; Harvard Apparatus). Healthy non-asthmatic and untreated (not received AA) mice were exposed to PBS aerosols to obtain the baseline Penh value, whereas asthmatic and AA-treated mice were exposed to a series of doubling concentrations of aerosolized methacholine (0.5, 1, 2, 4, and 8 mg/ml) to obtain AHR changes induced by treatments.

### Measurement of Cytokines Levels and WBCs in BAL Fluid

The number of two WBCs (neutrophils, an index of infection and Eos, indicating the level of asthma progression) in the BAL fluid were calculated as the percentage's values. In the other types of white blood cells, the neutrophil is important to determine the presence of infection in studied animals. The increased percentage of neutrophils show that there is infection and bronchial inflammation was initiated by infection, not by allergic reactions, thereby making the produced models worthless. The levels of IL-4, IL-5, IL-13, and INF-γ in BAL fluid were measured using Multi (bio)-Plex Pro™ Mouse Cytokine, Chemokine, and Growth Factor Assays (Bio-Rad, Nederland) as described before (22).

### IgE Levels and Eos in Serum

Before sacrificing the mice, blood samples were collected from the tail vein and the percentage of Eos was determined. Blood serum was separated and then total and OVA-specific IgE levels in serum were measured by ELISA, anti-Mouse IgE ELISA kit (BD Biosciences, USA and Cusabio Biotech, USA, respectively).

### Determining the Oxidative Stress Biomarkers

The oxidative stress indicators, including plasma level of MDA, pulmonary levels of superoxide dismutase (SOD), and catalase (CAT), were measured using the lung tissues. SOD level was calculated from the absorption reads at 412 nm for samples from the lung tissue (Thermofisher. USA), and the results were expressed as IU/mg protein. CAT content was evaluated by a colorimetric method (i.e., Goth method) which, as a brief explanation, was performed by incubating the lung tissue with H_2_O_2_ and then stopping the reaction by adding the AM. AM is a colorimetric indicator of the remaining H_2_O_2_ (it colors it to yellow) and the amount of H_2_O_2_ itself is directly associated with CAT degrading activity; the enzyme converts H_2_O_2_ to H_2_O and O_2_. The absorption of CAT experiments was read at 410 nm and the results were expressed as μmol H_2_O_2_/min/mg protein.

### Determining the Fibrotic Changes in Lung Tissue (Remodeling)

The hydroxyproline (HP) content in the lung tissue, as an important index of the deposition of collagen fibers, was measured by a colorimetric modified method introduced by Woessner (25). Lung tissue homogenates were hydrolyzed in 6 N HCL for 24 h at 120°C, then NaOH was added to the hydrolyzed samples for neutralization. Chloramine T reagent (1 mL, 0.05 M) was also added to the samples and maintained at room temperature for 20 min. For inactivation, 1.5 mL of 3.15 N perchloric acid was added. Afterwards, Ehlrich's solution was also added to the sample and incubated at 60°C for 20 min until the appearance of a reddish color. Absorbance was read at 560 nm and a standard curve drawn using pure HP was used to extrapolate the values of the HP content. The results were expressed as milligrams (mg) of HP per gram (g) of lung tissue (mg/g). Also, TGF-β was measured in supernatants of lung tissue homogenate (Abcam, US) as described before (22)

### Histopathology

At the end of each 2 weeks of AA-treatment period, the lung tissues of mice were isolated and maintained in formalin solution until fixation, then trimmed and paraffin-embedded. The sections were stained with Hematoxylin and Eosin (H&E), Alcian Blue, Alcian Blue-Periodic acid Schiff (PAS)-H&E, and Trichrome Masson stain. Afterwards, the perivascular and peribronchiolar inflammation were evaluated by using a routine scoring system (26). Goblet cell hyperplasia and mucus hyper secretion in the airways were determined using a point scoring system as described before (22, 26). The histopathological study was determined in 10 randomly selected microscopy fields on sections at 400x magnification by two pathologists. Goblet cell number was quantified per 100 epithelial cells at several randomly selected microscopy fields. The calculated number was Goblet Cell Index (GCI). The GCI was classified; score 0: GCI < 5%, score 1: 5% ≤ GCI < 25%, score 2: 25% ≤ GCI < 50%, score 3: 50% ≤ GCI < 75%, score 4: 75% ≤ GCI ≤ 100%. Moreover, eosinophils (Eos) population was examined on histological sections of lung tissue in five repeats. The absence or presence of a few Eos was scored 0, incomplete layer was scored 0.1–1, one complete layer of peribronchial/perivascular Eos was scored 1, two complete layers of peribronchial/perivascular Eos was scored 2, three complete layers of peribronchial/perivascular Eos was scored 3, and more than three complete layers of peribronchial/perivascular Eos was scored 4. Photomicrographs were taken with an Olympus B × 50 microscope equipped with a Leica DFC 320 digital Camera.

### Statistical Analysis

All experiments were repeated three times and results have been reported as a mean of three times. Data were expressed as means ± standard deviations (SD). Correlation analysis was carried out using Pearson's method. The paired *t*-test was used to analyze AHR methacholine changes from the baseline. The differences between asthmatic groups (AA-treated and non-treated) and control groups and the differences between AA–treated groups (asthmatic and non-asthmatic) and non-treated groups (not OVA- or AA-treated) were analyzed using unpaired Student's *t*-tests. Multiple comparisons were done using one-way ANOVA followed by Tukey's HSD tests. The *p value* at the level of <0.05 was supposed to be significant. SPSS (ver. 18) has been used for analysis and analyses were performed using GraphPad Prism (version 5.0).

### Ethics

This study was approved by the institutional review board (code number: 425/641).

## Results

### Airway Hyperresponsiveness

**Figure 2:**
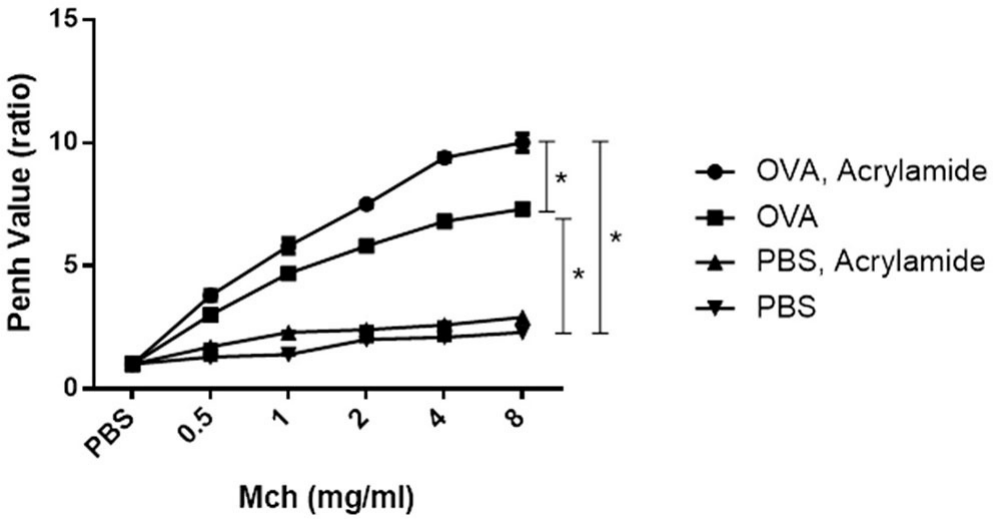
AHR in response to increasing doses of MCh by monitoring penh values. Mice were anesthetized and then tracheotomized. Asthmatic mice were exposed to a series of doubling concentrations of aerosolized MCH (0.5, 1, 2, 4, and 8 mg/ml) to obtain AHR changes induced by treatments. The penh values were increased in the OVA-sensitized and challenged mice compared with negative control mice for all concentrations of MCh. The OVA-sensitized and AA received group has maximum penh value in comparison with the OVA-sensitized group. The normal group (healthy group) has minimum penh values and the healthy AA received group has higher penh values in comparison with the normal group, but this increase was not significant (*P < 0.05).

The Penh values were significantly increased at all concentrations of MCh in the asthmatic mice compared to the negative control mice (*p* < 0.05) (Figure 2). Penh value in OVA-AA-treated group (at 4 mg/ml = 9.4 ± 0.15) is higher than OVA-sensitized (asthmatic) group (at 4 mg/ml = 6.8 ± 0.15) (*p* = 0.040). OVA-AA-treated group were significant different to OVA-sensitized and Penh values in the OVA-AA-treated group were higher than the asthmatic group in all concentrations. Penh value in healthy (non-asthmatic) AA-treated mice (at 4 mg/ml = 2.6 ± 0.08) were close to the levels in healthy untreated mice (at 4 mg/ml = 2.1 ± 0.10) indicating no significant change (*p* = 0.230). Other doses of MCh had similar patterns of 4 mg/ml, and all other paired groups had no significant differences between each other.

### Cell Counts

**Figure 3:**
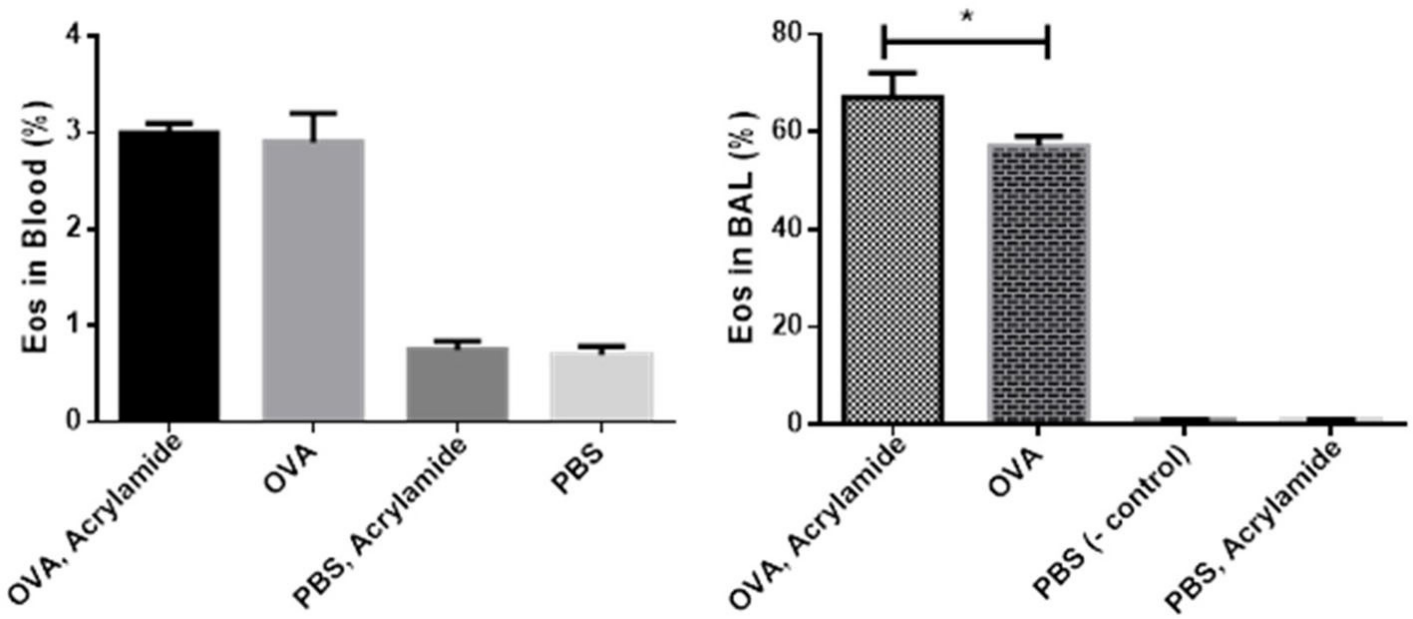
Percentage of Eos in Blood and BAL fluid. BAL fluid was collected, cytospined, and stained with Wright-Giemsa, and then the cells were counted. Eos in OVA-AA-group has the highest percentage in cytospined BAL and is significant from the OVA group. Eos percentage of blood WBC in the OVA-AA-group has no significant difference from the OVA group. Eos percentage in the healthy AA received group is similar to the healthy group in BAL and blood WBC (*P < 0.05).

**Figure 4:**
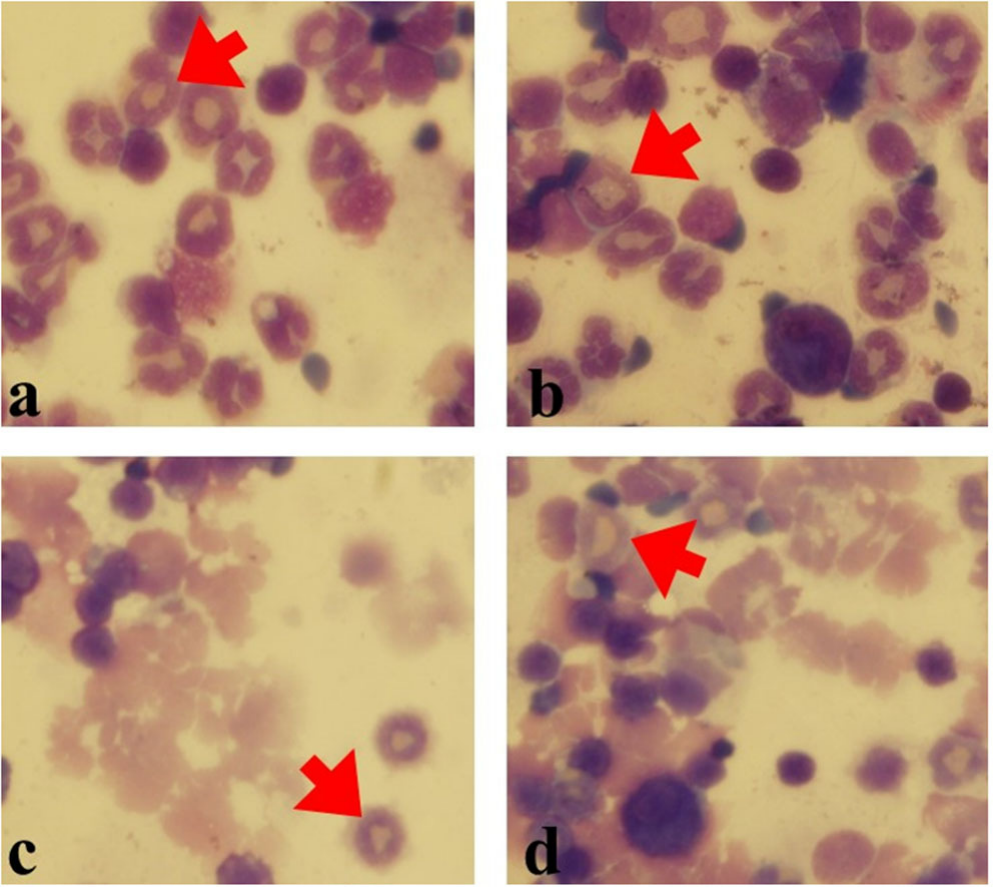
Eos in various groups (Gimsa staining). Eos percentage was counted in 200 cells. **(a)** OVA-AA-group, **(b)** OVA group, **(c)** healthy AA received group, **(d)** healthy group (red Arrows: Eos).

Eosinophils in BAL and blood slides were counted. The number of Eos in blood samples from OVA-AA-treated groups were not significantly different compared to the OVA-only treated (asthmatic) group (*p* = 0.880). However, the number of Eos in BAL samples from OVA-AA-treated groups were significantly different compared to the OVA-only treated (asthmatic) group (*p* = 0.019) (Figures 3, 4). In the AA-treated healthy group, no considerable increase in inflammatory responses was observed compared to the healthy and untreated group (*p* = 0.710). In all groups, the percentage of neutrophils in the BAL fluid were <4%, which shows that there was no infection in the studied animals and bronchial inflammation was produced by allergic reactions.

### IgE Levels

**Figure 5:**
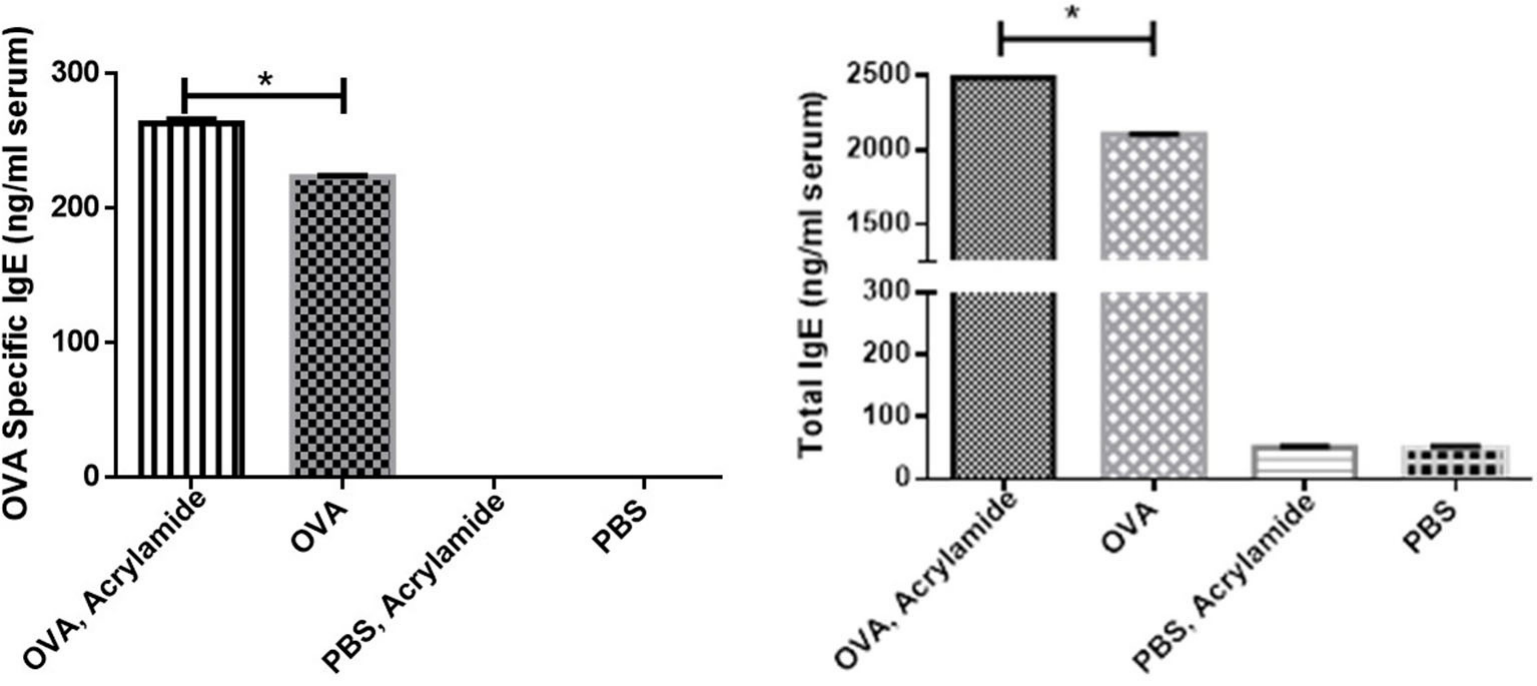
The total and OVA-specific IgE levels in serum of studied animals. Blood samples were collected, then total and OVA-specific IgE levels in serum were measured by ELISA. The OVA-specific and total IgE levels in serum of the OVA groups were increased. OVA-specific and total IgE levels in serum of the OVA-AA-group was increased significantly from the OVA group. OVA-specific and total IgE levels in serum of the healthy AA and healthy groups had no significant difference (*P < 0.05).

Total and OVA-specific IgE levels in serum were significantly increased in OVA- AA-received group (specific IgE: 263 ± 3, total IgE: 2,481 ± 7.5 ng/ml) compared to OVA-sensitized but not AA-treated group (asthmatic group) (specific IgE: 223 ± 1, total IgE: 2,100 ± 4.7 ng/ml) (*p* = 0.047, *p* = 0.049, respectively), at day 86. IgE levels in healthy (non-asthmatic) but not AA-treated group were close to the levels in the healthy untreated group and there was no significant difference between healthy and healthy AA-treated groups at day 86 (Figure 5).

### Cytokines Level in BAL Fluid

**Figure 6:**
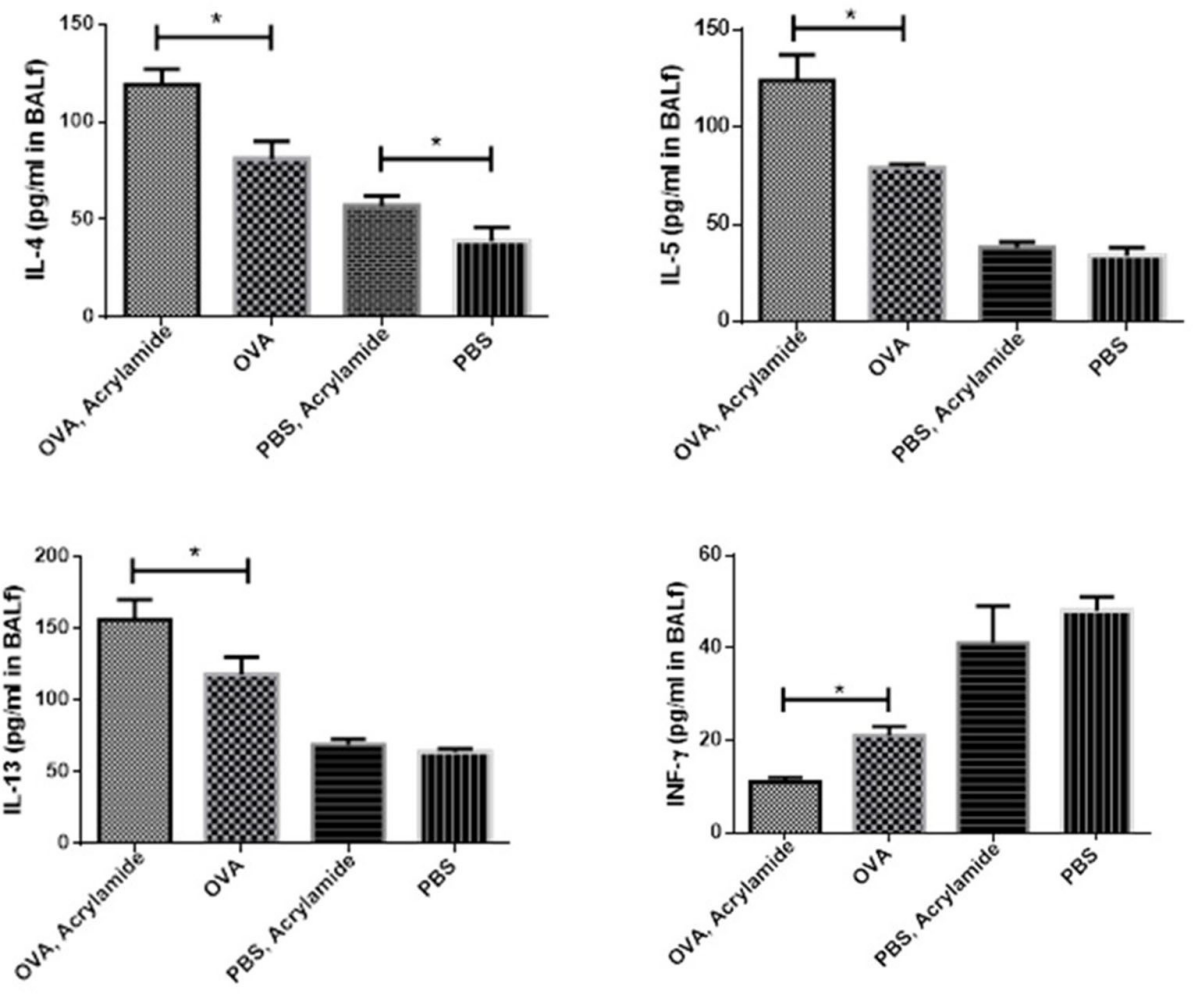
Cytokine levels in BAL of studied animals. The level of cytokines in BAL fluid were measured using Multi-Plex assays. The levels of IL-4, IL-5, and IL-13 in BAL fluid were increased in OVA groups as compared to healthy groups (PBS) and a reverse trend was found in IFN-γ level. IL-4, IL-5, and IL-13 levels in the OVA-AA-group were significantly increased in comparison to the OVA group. IFN-γ level in the OVA groups had no significant difference. The levels of IL-5, IL-13, and IFN-γ in healthy groups had no significant difference. IL-4 level in the healthy AA group was significantly higher than the healthy group (*P < 0.05).

The levels of IL-4, IL-5, and IL-13 were significantly increased in OVA-received groups compared to non-asthmatic groups (healthy groups), whereas a reverse trend was observed for IFN-γ levels (*p* < 0.05) (Figure 6). The levels of IL-4, IL-5, and IL-13 were significantly increased in the OVA-AA-received group (119 ± 8, 124 ± 13, and 156 ± 14 pg/ml, respectively), compared to the group that only received OVA (asthmatic groups) (81 ± 9, 79 ± 2, and 118 ± 12 pg/ml, respectively) (*p* = 0.014, 0.007, 0.024, respectively). The levels of IL-4, IL-5, IL-13, and IFN-γ had no significant difference between the healthy AA-received group and the healthy group (Figure 6).

### The Oxidative Stress Biomarkers

**Figure 7:**
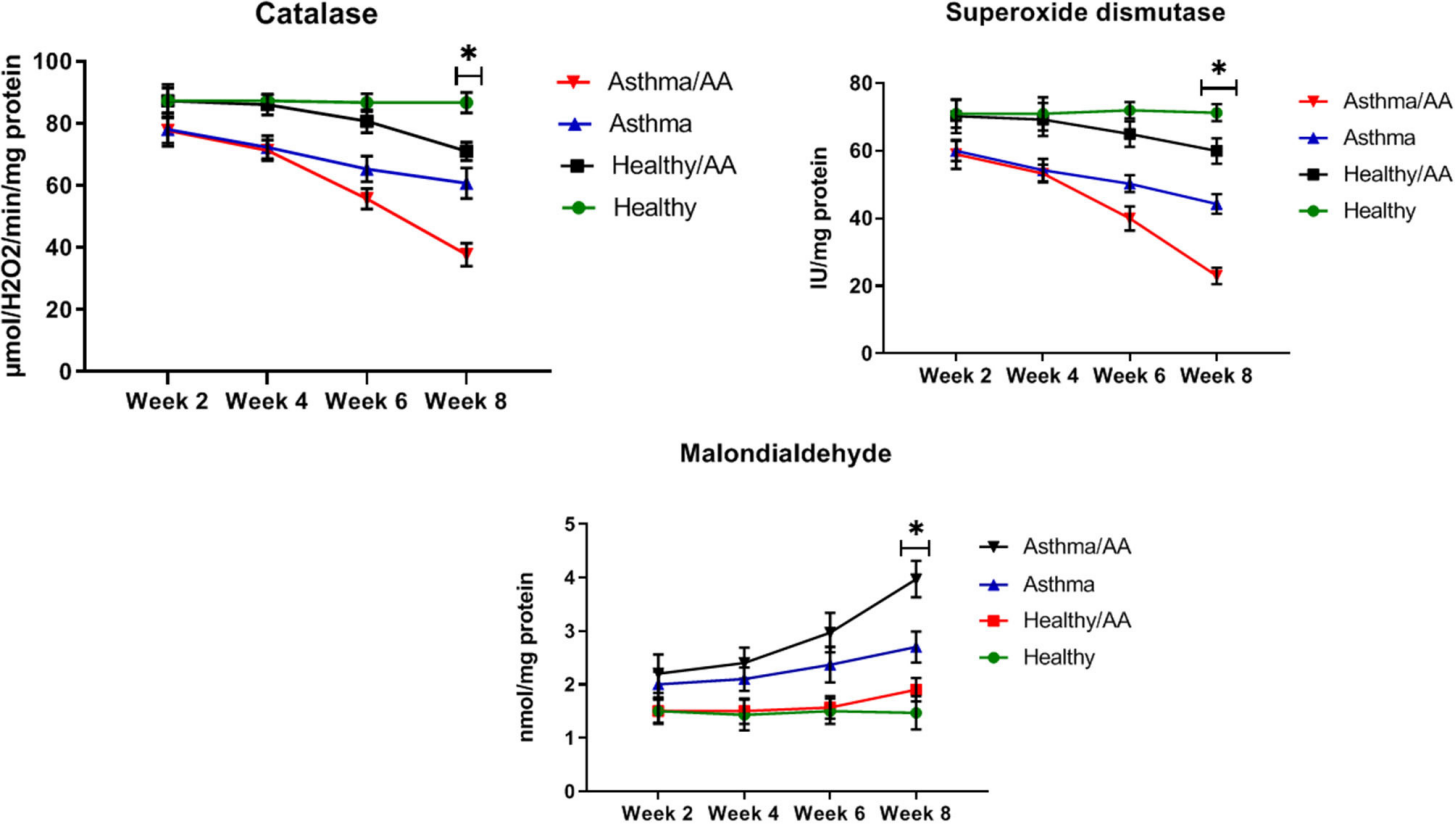
The SOD, CAT, and MDA. The oxidative stress indicators were measured using the lung tissues. SOD level was calculated from the absorption reads at 412 nm for samples from the lung tissue, and the results were expressed as IU/mg protein. CAT content was evaluated by a colorimetric method by incubating the lung tissue with H_2_O_2_. The absorption of CAT experiments was read at 410 nm and the results were expressed as μmol H_2_O_2_/min/mg protein. The SOD, CAT, and MDA levels in all groups have been shown at four time points (weeks 2, 4, 6, and 8). In the 8th week, SOD levels had a significant difference between the asthma group and asthma AA received group and it was decreased in the asthma AA received group. In the other groups, there was no significant difference. The CAT in week 8 had significant difference between the asthma group and asthma AA received group. In the second week, MDA in the asthma AA received group was increased significantly in comparison with the asthma group and in the 8th week, MDA was increased significantly in the asthma AA received group compared with the asthma group. *In this week, the groups have significant difference in compare paired groups (healthy with healthy/AA, asthma with asthma/AA).

The oxidative stress indicators were measured in the studied groups. SOD levels after 2 weeks had no significant different between the asthma group and the asthma AA-received group (60.0 ± 2.94 and 59.0 ± 4.32 IU/mg protein, respectively). But at 8 weeks, SOD levels had a significant difference between the asthma group (44.33 ± 2.87 IU/mg protein) and asthma AA-received group (*p* < 0.05) (23.00 ± 2.45 IU/mg protein), and it was decreased in the asthma AA-received group. Similarly, the CAT after 2 weeks had no significant difference between the asthma group (78.0 ± 5.35 μmol H_2_O_2_/min/mg protein) and asthma AA-received group (77.7 ± 4.11 μmol H_2_O_2_/min/mg protein). At week 8, CAT showed a significant difference between the asthma group and asthma AA-received group and it was decreased in the asthma AA-received group (60.67 ± 4.92 and 37.67 ± 3.68 μmol H_2_O_2_/min/mg protein, respectively) (*p* < 0.05). In the 2nd week, MDA in the asthma AA-received group (2.2 ± 0.36) was increased significantly when compared with the asthma group (2 ± 0.24) and In the 8th week (*p* < 0.05), MDA was increased significantly in the asthma AA-received group compare with the asthma group (*p* < 0.05) (3.97 ± 0.34 and 2.70 ± 0.29, respectively) (Figure 7).

### The Fibrotic Factors

**Figure 8:**
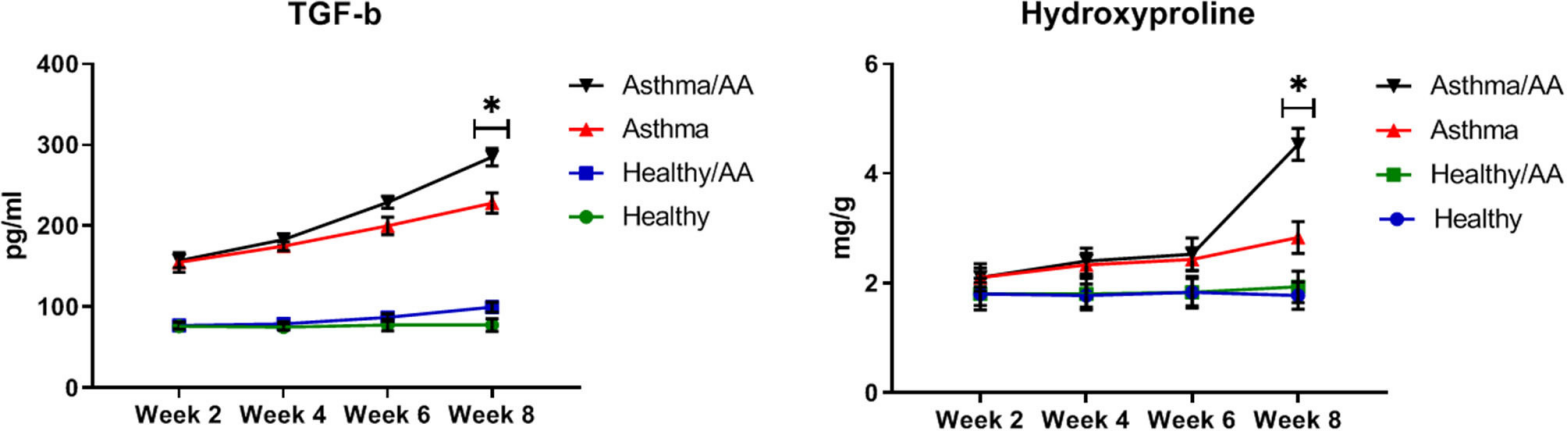
The remodeling factors. The hydroxyproline (HP) content in lung tissue as an important index of deposition of collagen fibers was measured by a colorimetric modified method. Absorbance was read at 560 nm. The results were expressed as milligrams (mg) of HP per gram (g) of lung tissue (mg/g). Also, TGF-β was measured in supernatants of lung tissue homogenate. The changes of HP and TGF-β in all groups have been shown at four time points (weeks 2, 4, 6, and 8). HP and TGF-β have significant changes in two asthma group and asthma AA received group in the 8th week. *In this week, the groups have significant difference in compare paired groups (healthy with healthy/AA, asthma with asthma/AA).

HP and TGF-β, as two main factors in the fibrosis of bronchial and airway remodeling in asthmatic patients, showed significant changes in two asthma groups and the asthma AA-received group in the 8th week (*p* < 0.05) (HP; 2.83 ± 0.29 and 4.53 ± 0.29 mg/g, respectively, TGF-β; 228.33 ± 12.47 and 285.00 ± 10.80 pg/ml, respectively) (Figure 8).

### Histopathology

**Figure 9:**
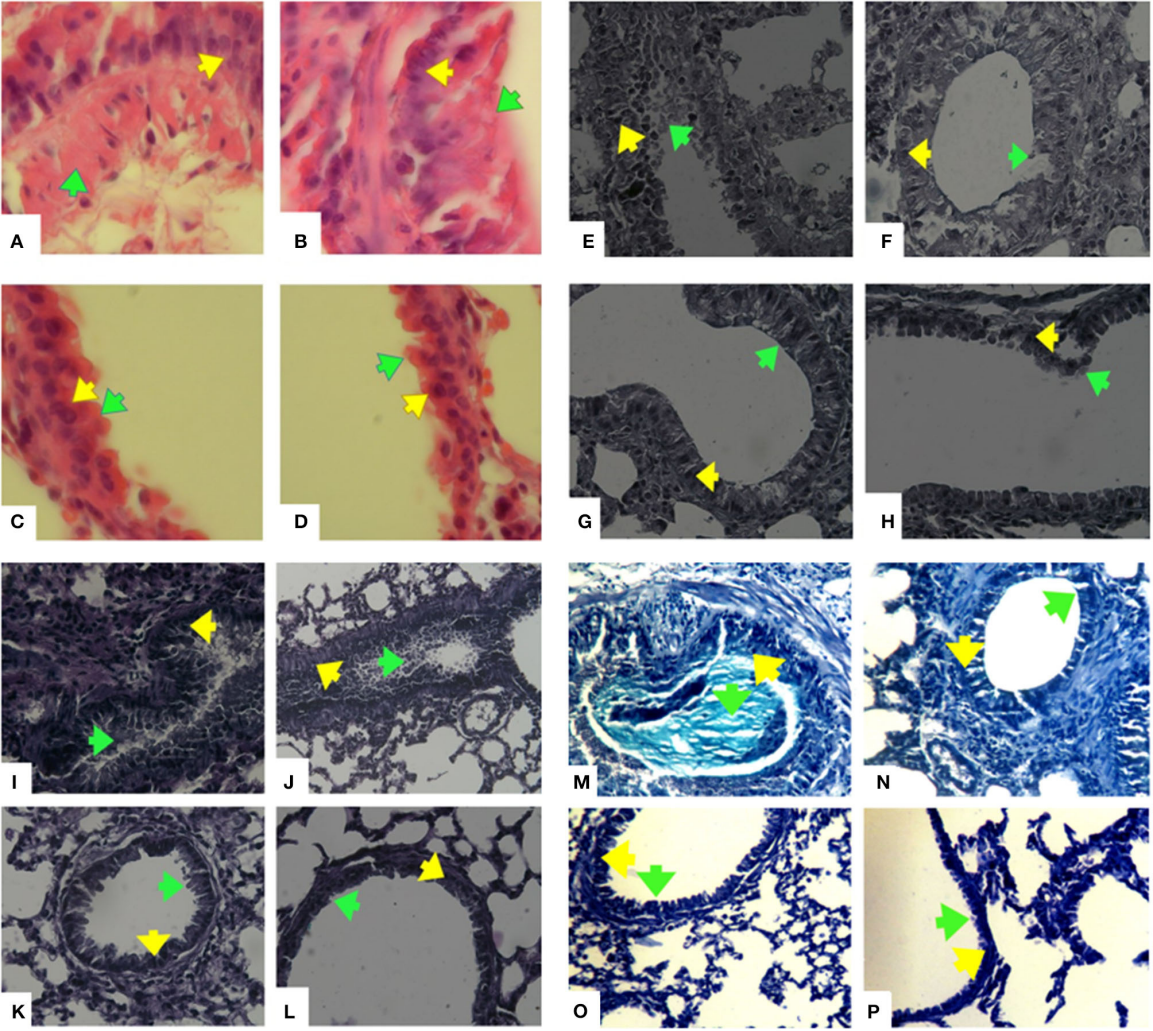
Histopathology of lung sections. Lung tissues were fixed then stained with H&E, Alcian Blue, Alcian Blue- PAS-H&E, and Trichrome Masson stain. Afterwards, the perivascular, peribronchiolar inflammation (eosinophilic inflammation), goblet cell hyperplasia, and mucus hyper secretion were evaluated in 10 randomly selected microscopy fields on sections at 400 x magnification. Hematoxilin-Eosine (H&E) stained lungs of mice, **(A)** OVA-AA-group, **(B)** OVA group, **(C)** healthy AA received group, **(D)** healthy group. Goblet cell hyperplasia and mucus hypersecretion in OVA groups have been increased and the OVA-AA-group have a higher score than the OVA group. Alcian Blue stained lungs of mice, **(E)** OVA-AA-group, **(F)** OVA group, **(G)** healthy AA received group, **(H)** healthy group. Goblet cell hyperplasia and mucus hypersecretion in OVA groups have increased and the OVA-AA-group has a higher score than the OVA group. Alcian Blue-Periodic acid Schiff (PAS)-H&E stained lungs of mice, **(I)** OVA-AA-group, **(J)** OVA group, **(K)** healthy AA received group, **(L)** healthy group. Goblet cell hyperplasia and mucus hypersecretion in OVA groups have increased and the OVA-AA-group have a higher score than the OVA group. Trichrome Masson stained lungs of mice, **(M)** OVA-AA-group, **(N)** OVA group, **(O)** healthy AA received group, **(P)** healthy group. Initiation of remodeling in the OVA-AA-group was increased compared to the OVA group. Green Arrows show mucus hypersecretion and yellow Arrows show goblet cells-hyperplasia.

**Figure 10:**
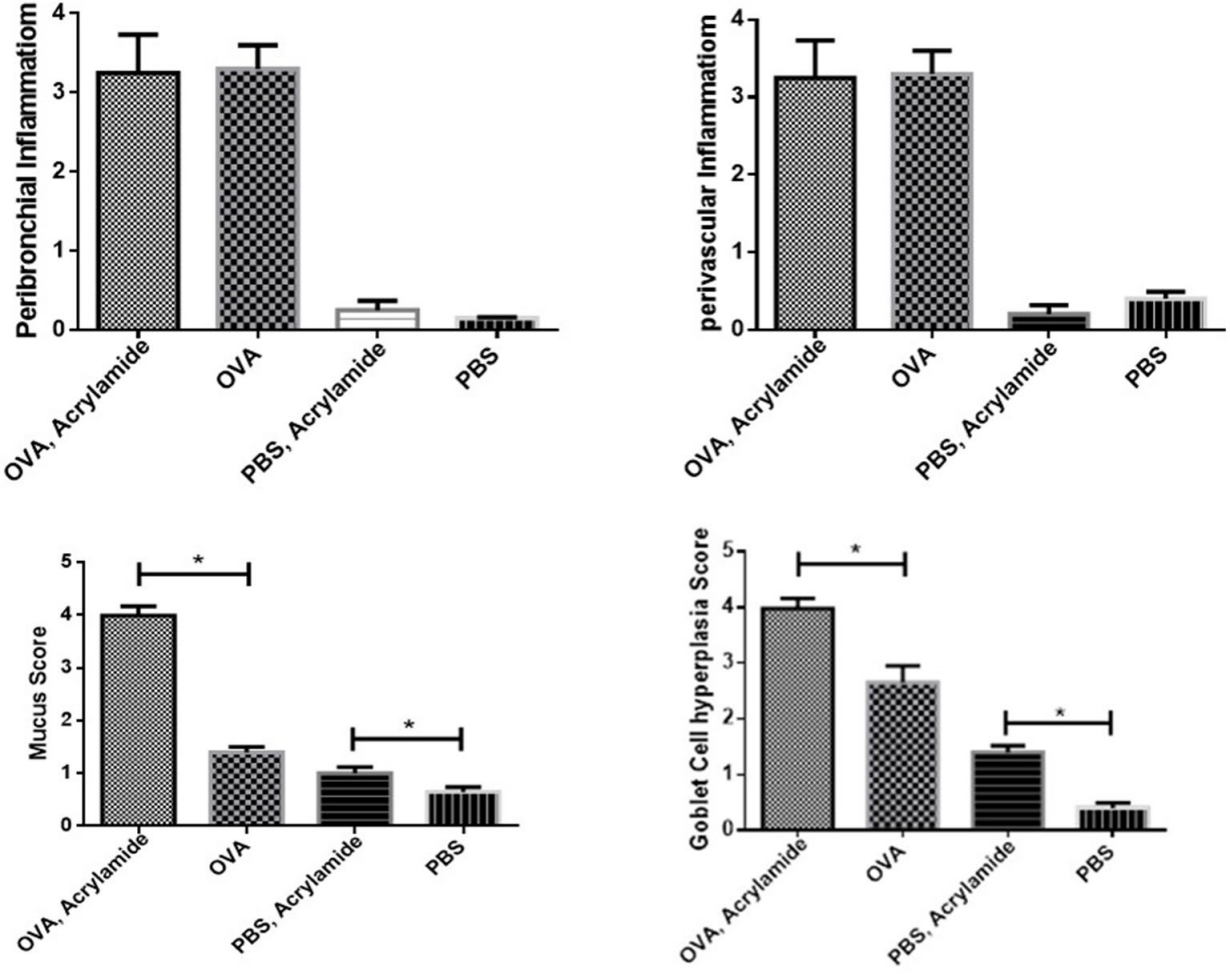
Inflammatory cells infiltration (Eosiniphilic inflammation) around lung bronchioles and vascular. Inflammation was increased in OVA groups as compared to normal groups. OVA groups showed no significant difference and healthy groups had no significant difference. Mucus hypersecretion and Goblet cell hyperplasia. Mucus secretion and goblet cell hyperplasia were increased in OVA groups as compared to healthy groups. OVA-AA-group showed significantly higher mucus secretion and goblet cell hyperplasia than the OVA group. The healthy AA group has significantly higher mucus hypersecretion and goblet cell hyperplasia than the healthy group (*P < 0.05).

Mucus hyper secretion and goblet cell hyperplasia were significantly induced in the airways of OVA-challenged mice (asthmatic groups) compared to PBS-challenged mice (healthy groups) (*p* < 0.05). Mucus hyper secretion and goblet cell hyperplasia were significantly induced in the airways of OVA-challenged mice that also received AA (asthma AA-received group) compared to the group only treated by OVA (asthmatic group) (*p* < 0.048) (Figures 9, 10). Histology revealed pathologic features of asthma in the OVA-challenged mice that also received AA compared to the group only treated with OVA. Moreover, an increased number of inflammatory cells in the lungs of the asthmatic mice (eosinophilic inflammation) were observed in comparison to the PBS-challenged groups (*p* < 0.018). Inflammation in the PBS-challenged AA-treated group was at a similar level to that in the PBS-challenged but untreated group (Figures 9, 10).

## Discussion

The oral LD_50_ of AA was reported at 107 mg/kg body weight (b.w.) for mice (24) and the benchmark dose level that is linked with a 10% extra risk of side effects in the exposed test animals (BMDL_10_) was reported at 0.43 mg/kg b.w. per day for peripheral neuropathy in rats and at 0.17 mg/kg b.w. per day for neoplastic effects in mice (24).

In the present study, we found that AA acts as an allergen in the airways and induces strong inflammatory responses in asthmatic mice and increases allergic reactions that lead to the progression of a moderate primary-stage asthma to a severe late-stage complicated asthma. Although we could not find significant inflammatory changes in healthy non-asthmatic airways by AA poisoning, we could observe harmful effects induced by AA in pre-sensitized asthmatic mice and that AA has an effect on sensitized airways. The OVA-AA-treated group showed more severe symptoms of allergic asthma than that seen in the OVA-only treated group (asthmatic group). The symptoms seen in the non-asthmatic but AA-treated group was at a similar level to that seen in the healthy untreated group.

Acrylamide is toxic for cells and has a poisoning effect on tissues. It is used industrially as a monomer precursor for the production of dyes and polymers such as sodium dodecyl sulfate polyacrylamide gel (27, 28). AA in fried and baked foods is an active health-threatening toxic factor. Plants are an important source of AA contamination of food (28).

AA is found mainly in vegetarian foods and potato chips, which contain the highest levels of AA. Bread also contains high levels of AA (23). In this study, we have evaluated the effect of AA toxicity in an animal model of allergic asthma. We used a dose of AA in pelleted food for mice similar to that in potato chips.

In asthmatic mice, AHR (Penh value) was increased compared to in negative control mice. The level of AHR was higher in the OVA-AA-treated group in comparison to the OVA-only treated group (asthma group). The non-asthmatic AA-treated group showed no change in AHR level compared to the healthy untreated group. These results showed that AA worsens the inflammatory responses in asthmatic mice, but has little or no effect in healthy mice. Previous studies showed that AA can act as an allergic agent and cause a cutaneous anaphylaxis reaction, atopic dermatitis, eczema, and allergic reactions (29–31). In addition, we found that the total and OVA-specific IgE levels in the serum of the OVA-AA-treated group was increased in comparison to the OVA-only treated group (asthma group), whereas the levels in the healthy (non-asthmatic) AA-treated group were close to the levels in the healthy untreated group. Moreover, increased levels of type-2 cytokines and decreased levels of type-1 cytokines in the OVA-AA-treated group were significantly changed in comparison to the OVA-only treated group (asthma group), but the non-asthmatic AA-treated group was in a similar condition to the healthy untreated group. Our results indicate that when AA is present in food it can act as a strong triggering agent for the worsening of allergic responses and inflammation in the airways. It can exacerbate the inflammatory symptoms of allergic asthma and lead to a severe complicated form of allergic asthma.

DeGrandchamp et al. (32), have found that a single dose of AA triggers pathological lesions and remodeling in the nervous system. AA as a food contaminant is formed during the high-temperature preparation of carbohydrate-rich food (32). Stošić et al. (33) determined that AA in adult male Wistar rats leads to the remodeling of Langerhans islets and decreases cells' volume density (33). We found that healthy mice that only received AA were in similar conditions to healthy untreated mice, but asthmatic AA-treated mice ha significant changes in airway remodeling-associated biomarkers. These biomarkers include HP and TGF-β, which are two fibrotic factors involved in remodeling in asthmatic animals, which both increased significantly in the asthma AA-received group in comparison with the asthma group in the 8th week. Remodeling is a time-consuming process, therefore, increasing levels of TGF-β and HP were observed only after a long time (after 8 weeks). In the Sarocka et al. (34) study, increased endocortical remodeling and higher levels of serum calcium associated with AA administration were reported (34). In our study, the OVA-AA-treated mice also experienced worsened levels of oxidative stress indicators. After 8 weeks, SOD and CAT levels were decreased significantly in the asthma AA-received group when compared with the asthma group. These results showed that protective factors in asthmatic bronchial with AA presence have been inactivated and harmful factors have been increased over a long time and injured the airway tissue, which leads to remodeling and other pathological changes in the airway. Remodeling initiation was not observed after 2 weeks, but after 8 weeks, histopathology sections start showing remodeling in OVA-AA-treated bronchi.

Eos is the predominant cell in BAL fluid and blood of asthmatic patients. In our study, the Eos number in BAL and the blood of OVA- AA-treated groups were higher than that in OVA-sensitized groups. Moreover, we found that AA can induce severe eosinophilic inflammation in asthmatic airways. Zaidi et al. in 1994, showed that AA causes immunotoxicity and reduction of circulating blood lymphocytes (35). In addition, they found that AA suppresses the humoral and cellular immunity that leads to weight loss of the spleen, thymus, and mesenteric lymph nodes. The toxic effects of AA were completely or partially reversed by treatment with sixth mycelial fraction acetone, a fungal origin interferon inducer (36). In our study, the peribranchial and perivascular inflammation, mucus hyper secretion, and goblet cell hyperplasia were significantly increased in the airways of the OVA-only stimulated group (asthma group) compared to the healthy untreated group. Inflammation was more severely induced in the OVA-AA-treated group compared to control groups. Considering these results, we can hypothesize that AA should be the central factor causing asthmatic exacerbations in atopic patients who consume foods contaminated by AA. Also, AA can worsen the allergic inflammatory responses in asthmatic patients.

AA can induce allergic reactions in the case of cutaneous contact. In addition, other types of AA-cell contact, e.g., subcutaneous administration, can also cause hepatotoxic effects and can induce the degeneration of cells in the liver, lung, blood, and other tissues (36, 37). Morin hydrate, a polyphenolic compound from the Moraceae family of plants, is one of the natural protectants against AA (38, 39). Sixth mycelial fraction acetone (6-MFA) is another natural anti-AA compound derived from a mycelial fungus named *Aspergillus ochraceus* that prevents AA toxicity (36). These may be candidates for the treatment of AA poisoning, especially in asthmatic patients.

AA as a dermal allergen can trigger allergic asthma in atopic patients. However, on the other hand, some derivatives of AA can inhibit the release of histamine, allergic mediators, and the activation of 5-lipoxygenase (39, 40). AL-3264 (N-[4-[4-(diphenylmethyl)-1-piperazinyl]butyl]-3-(6-methyl-3- pyridyl) acrylamide, an AA derivative, showed promising anti-allergic effects in experimental animal models of asthma and could inhibit antigen-induced bronchoconstriction (41, 42). Therefore, AL-3264 or other similar AA-derived compounds can be used as antidotes to compete with AA to reverse its toxic effects in asthmatic patients. On the other hand, some studies showed that there are some antioxidant compounds in plants with protective effects against various toxicants (43, 44).

However, there are several limitations in our study that should be considered for future studies on the toxic effects of AA-contaminated foods in asthmatic patients. First, we did not evaluate the toxic effect and allergenicity of poly AA administered by inhalation in allergic asthma. We just examined its effects due to food contamination administered orally. Next, we used AA in food in just one dose and did not examine its effect in different doses. Lastly, we used an animal model to study the toxic effects induced by AA, so it should also be studied in human subjects in future studies. We also could not determine the level of IgG1 and IgG2a, important immunoglobulins, in this study.

In conclusion, in the present study we found that AA can be a harmful worsening agent in allergic asthma and can increase the severity of inflammatory responses in asthmatic mice. It can also induce the progression of mild asthma to a severe form of asthma and subsequently to an uncontrolled and complicated form of asthma. Therefore, prevention of AA exposure is strongly recommended for patients with asthma. Asthmatic patients should pay more attention to their dietary habits and carefully choose the foods in their dietary regimen and remove AA containing foods (potato chips, bread, and other high AA-containing foods) from their dietary regimen.

## Data Availability Statement

The raw data supporting the conclusions of this article will be made available by the authors, without undue reservation.

## Ethics Statement

The animal study was reviewed and approved by The studies involving animals were reviewed and approved by ethics committee of the Shahid Sadoughi University of Medical Sciences.

## Author Contributions

BH participated in the design of the study. SMA assisted in the establishment of airway hyperresponsiveness test and statistical analysis. MA supervised the study. GV assisted in result analysis and drafting the manuscript. SSA participated in the design of the study, animal works, lab analysis, and drafting the manuscript. This study is the outcome of the postdoc program of the SSA, who is one of the authors. All authors contributed to the article and approved the submitted version.

## Conflict of Interest

The authors declare that the research was conducted in the absence of any commercial or financial relationships that could be construed as a potential conflict of interest.

## Abbreviations

AA: acrylamide
AHR: Airway hyperresponsiveness
AM: ammonium molybdate
BAL: broncho-alveolar lavage
CAT: catalase
Eos: eosinophil
H&E: Hematoxylin and Eosin
HP: hydroxyproline
IgE: immunoglobulin E
IL: interleukin
INF: interferon
MCh: methacholine
MDA: malondialdehyde
OVA: ovalbumin
SD: standard deviations
SOD: superoxide dismutase
TGF: transforming growth factor
Th: Lymphocyte T Helper
WBC: white blood cell.
